# Harnessing Plasma Biomarkers to Predict Immunotherapy Outcomes in Hepatocellular Carcinoma: The Role of cfDNA, ctDNA, and Cytokines

**DOI:** 10.3390/ijms26062794

**Published:** 2025-03-20

**Authors:** Elena Vargas-Accarino, Mónica Higuera, María Bermúdez-Ramos, Agnès Soriano-Varela, María Torrens, Mònica Pons, Ana María Aransay, José Ezequiel Martín, Francisco Rodríguez-Frías, Xavier Merino, Beatriz Mínguez

**Affiliations:** 1 Liver Cancer Research Group, Liver Diseases, Vall d’Hebron Institut de Recerca (VHIR), Vall d’Hebron Barcelona Hospital Campus, 08035 Barcelona, Spain; elena.vargas@vhir.org (E.V.-A.); monica.higuera@vhir.org (M.H.); mbermudezr.germanstrias@gencat.cat (M.B.-R.); agnes.soriano@vhir.org (A.S.-V.); maria.torrens@vallhebron.cat (M.T.); 2 Department of Medicine, Campus de la UAB, Universitat Autònoma de Barcelona (UAB), Bellaterra, 08193 Cerdanyola del Vallès, Spain; 3 Centro de Investigación Biomédica en Red de Enfermedades Hepáticas y Digestivas (CIBERehd), Instituto de Salud Carlos III, 28029 Madrid, Spain; monica.pons@vallhebron.cat (M.P.); frarodri@gmail.com (F.R.-F.); 4 Liver Unit, Hospital Universitario Vall d’Hebron, Vall d’Hebron Barcelona Hospital Campus, 08035 Barcelona, Spain; 5 Genome Analysis Platform, CIC bioGUNE, 48160 Derio, Spain; amaransay@cicbiogune.es (A.M.A.); jmartin@cicbiogune.es (J.E.M.); 6 Microbiology and Biochemistry Department, Hospital Universitario Vall d’Hebron, Vall d’Hebron Barcelona Hospital Campus, 08035 Barcelona, Spain; 7 Radiology Department, Hospital Universitario Vall d’Hebron, Vall d’Hebron Barcelona Hospital Campus, 08035 Barcelona, Spain; xavier.merino.idi@gencat.cat

**Keywords:** hepatocellular carcinoma, immunotherapy, liquid biopsy, cfDNA, ctDNA, biomarkers

## Abstract

Immunotherapy has improved survival in patients with advanced hepatocellular carcinoma (HCC); yet, objective radiological responses occur in only about 20% of cases, suggesting variable benefits. This study aimed to identify serologic markers predictive of response to immune checkpoint inhibitors (ICIs). A cohort of 38 advanced HCC patients receiving immunotherapy was prospectively analyzed. Levels of cell-free DNA (cfDNA), circulating tumor DNA (ctDNA), and cytokines were measured pre-treatment and three months post-treatment initiation. Genomic profiling of ctDNA was also conducted. Baseline levels of cfDNA and ctDNA effectively discriminated HCC patients based on their radiological response to ICIs. Additionally, individuals with pathologic mutations in the *CDKN2A* gene exhibited significantly reduced survival. Patients with progressive disease (PD) as their best radiological response had significantly fewer copy number variations (CNVs) than those with a radiological response. Furthermore, levels of IL10, PD1, and TGFβ assessed after three months of treatment showed significant variations correlating with survival status. In conclusion, the analysis of cfDNA, ctDNA, and cytokines may improve treatment selection for HCC patients by predicting their expected response to immunotherapies.

## 1. Introduction

Hepatocellular carcinoma (HCC) ranks as the second leading cause of cancer-related mortality worldwide, with its incidence continuing to rise globally [1]. Despite the implementation of screening programs in patients with cirrhosis, fewer than 50% of patients are diagnosed at an early stage, when curative treatments can be applied [2].

Over the past decade, systemic therapies for HCC have undergone rapid evolution [2], with immune checkpoint inhibitors (ICIs) dramatically transforming the therapeutic landscape for advanced cases. Monotherapy with the anti-PD-1 antibodies nivolumab and pembrolizumab showed objective response rates of 15–20%, leading to their accelerated approval by the U.S. Food and Drug Administration. However, phase III clinical trials failed to meet their primary endpoints [3,4,5]. In contrast, the combination of the anti-PD-L1 agent atezolizumab and the anti-vascular endothelial growth factor monoclonal antibody bevacizumab has demonstrated a significant improvement in overall survival (OS) compared to the previous standard of care, sorafenib, leading to its approval as a first-line systemic treatment for patients with unresectable HCC [6]. This combination not only offers enhanced survival benefits but also represents a shift in the treatment paradigm, moving away from monotherapy with tyrosine kinase inhibitors to more targeted immunotherapy approaches. More recently, the combination of the anti-PD-L1 antibody durvalumab with the anti-Cytotoxic T-Lymphocyte Antigen 4 (CTLA-4) antibody tremelimumab has become available for similar patient profiles [7], further expanding the therapeutic options and underscoring the critical role of combination therapies in managing advanced HCC [2,3,4,5,6,7].

Survival is widely recognized as the primary endpoint in clinical trials for cancer therapeutics, establishing the ultimate measure of a treatment’s efficacy. Nevertheless, the identification of secondary endpoints or surrogate markers is crucial for evaluating therapeutic activity and potential patient benefits in a more timely and efficient manner. Radiological response, historically adopted as a surrogate marker in oncology trials, has been instrumental in correlating tumor shrinkage with OS, particularly within the context of traditional chemotherapeutic approaches. This correlation has provided a foundational framework for assessing treatment efficacy in a variety of malignancies. However, the applicability of radiological response as a surrogate marker varies with different classes of therapies, such as tyrosine kinase inhibitors and, more recently, immunotherapies, which may not exhibit the same direct correlation between tumor reduction and survival. Despite these challenges, radiological response continues to serve as a valuable and quantifiable endpoint in clinical settings. It offers clinicians a tangible measure of treatment impact and remains integral to the ongoing assessment and refinement of therapeutic strategies in oncology. Consequently, exploring additional surrogate markers alongside radiological response is imperative to capture the full spectrum of therapeutic effects and to guide clinical decision-making processes more effectively [8].

Objective radiological response (ORR) to ICIs has been observed in only approximately 20–30% of patients with HCC [8]. This relatively modest response rate underscores the critical importance of identifying patients who are most likely to benefit from these therapies. By doing so, clinicians can optimize the use of ICIs, ensuring that their administration is both rational and efficient while also minimizing unnecessary exposure to potential side effects for those patients who are less likely to respond favorably.

In pursuit of this goal, a variety of potential biomarkers have been investigated to assess the efficacy of ICIs across different cancer types, including tumor-infiltrating lymphocytes, programmed cell death ligand (PD-L1) expression, and tumor mutational burden (TMB) [9,10].

However, in the context of HCC, the anticipated correlations between changes in PD-L1 expression and clinical outcomes such as tumor response or progression-free survival (PFS) have not been conclusively demonstrated [11]. In particular, the expression of PD-L1 has not shown a significant association with treatment response to ICIs, such nivolumab [3], pembrolizumab [9] or atezolizumab, in combination with bevacizumab [12], in HCC patients. Additionally, although *CTNNB1*-activating mutations have been suggested to contribute to immune escape and resistance to anti-PD-1 therapies [13], the relevance of this association in patients treated with atezolizumab/bevacizumab [14] or nivolumab [5] remains unclear and warrants further investigation. Alpha-fetoprotein (AFP) is the most commonly studied serological marker for HCC and has also been evaluated as a serological marker in immunotherapy. Patients who exhibit a significant reduction in AFP levels early in treatment tend to experience longer median OS and PFS [15], as well as higher overall response rates [16]. However, AFP levels may not be elevated in all patients, limiting its utility as a biomarker. Its effectiveness is restricted because it does not provide information at the onset of treatment and requires subsequent assessment after treatment initiation.

It has been hypothesized that tumor-infiltrating lymphocytes (TILs) may be indicative of the effectiveness of immune checkpoint inhibitors [17]. In the HCC setting, the presence of CD3+ and CD8+ T cells has been associated with a trend towards longer OS in patients enrolled in the CheckMate-040 study [18].

The primary objective of this study was to determine whether levels of plasma cell-free DNA (cfDNA), circulating tumoral DNA (ctDNA), and cytokines, in conjunction with ctDNA mutational profiling, are associated with the response to ICIs in patients with unresectable HCC. To address this objective, we conducted a prospective study involving 38 patients undergoing immunotherapy, systematically collecting plasma samples at the start of treatment and after three months, providing critical longitudinal data to explore these potential biomarkers in relation to treatment outcomes.

## 2. Results

### 2.1. Patient Characteristics

Thirty-eight patients with unresectable HCC were prospectively recruited and treated with ICIs at the Liver Unit of Hospital Universitari Vall d’Hebron. The median age of the patients was 68.7 years, with a predominance of males (81.6%). Advanced fibrosis or cirrhosis was present in 69.3% of the cohort. The median follow-up duration was 16.5 months, ranging from 2 to 60 months. Viral etiology was identified as the underlying cause in 60.6% of the patients, with hepatitis C virus infection being the most common, accounting for 55.3% of cases. A significant majority, 78.9%, were classified at the Barcelona Clinic Liver Cancer (BCLC) C stage. At the start of ICI therapy, 44.7% of patients had previously received systemic therapy with tyrosine kinase inhibitors (TKIs), while for 57.9% of patients, ICI represented their first systemic treatment. At treatment onset, 84.2% were categorized as Child-Pugh A and exhibited an Eastern Cooperative Oncology Group (ECOG) performance status of 0. Detailed clinicopathological characteristics are presented in Table 1.

When analyzed based on survival status, no differences were observed in baseline alpha-fetoprotein (AFP) levels among the patients. However, three months after initiating treatment, patients who did not survive exhibited significantly higher AFP levels (157.5 [8.7, 7875] vs. 3.9 [2, 7.2], *p* = 0.008). Additionally, a notable increase in AFP levels was observed when comparing baseline levels with those at the three-month mark post-ICI initiation (3.7 [−0.2, 726.3] vs. −2.1 [−10.7, 0], *p* = 0.004).

Non-surviving patients also exhibited higher baseline gamma-glutamyl transferase (GGT) levels (189 [92, 282] vs. 80 [40, 134], *p* = 0.018) and lower baseline hemoglobin levels (12.5 [11.6, 13.6] vs. 14 [13.3, 15], *p* = 0.03). No other clinical differences were observed according to survival status (Supplementary Tables S1 and S2).

**Figure 1:**
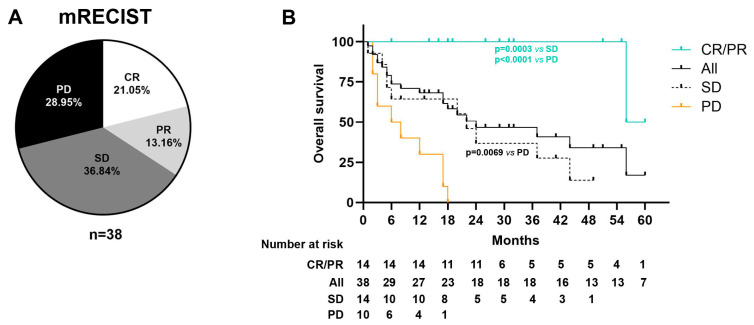
Clinical outcomes. (**A**) Best radiological response assessed by mRECIST. (**B**) Kaplan–Meier curve of overall survival. CR: complete response, PR: partial response, SD: stable disease, PD: progressive disease, mRECIST: Modified Response Evaluation Criteria in Solid Tumors.

Throughout the follow-up period, tumor progression was observed in 24 patients (63.2%), and 21 patients (55.3%) died, with 19 of them (90.5%) having experienced tumor progression (Table 1). According to Modified Response Evaluation Criteria in Solid Tumors (mRECIST), among the 38 patients enrolled, 8 (21.1%) achieved complete response (CR), 5 (13.2%) achieved partial response (PR), 14 (36.8%) had stable disease (SD), and 11 (28.9%) experienced progressive disease (PD) as their best radiological response (Figure 1A). Kaplan–Meier survival analysis revealed that patients with CR or PR exhibited a statistically significant higher median OS compared with those with SD or PD (58, 24, and 7 months, respectively) (*p* = 0.0003 for CR/PR vs. SD, and *p* < 0.0001 for CR/PR vs. PD). The median PFS for the entire cohort was 10 months, while the median OS was 24 months (Figure 1B).

The type of radiological response, as assessed by mRECIST, showed statistically significant differences in OS outcomes among patients categorized as having a complete response (CR), partial response (PR), stable disease (SD), or progressive disease (PD). When comparing survivors and non-survivors, the rates of CR were 47.1% and 0%, PR were 23.5% and 4.8%, SD were 23.5% and 47.6%, and PD were 5.9% and 47.6%, respectively (*p* < 0.001). These findings support the idea that radiological response, as measured by mRECIST, could act as a surrogate marker for predicting OS.

### 2.2. Immune-Related Adverse Events (irAEs)

The overall incidence of irAEs among the study cohort was 50% (19 out of 38 patients). A detailed summary of the representative irAEs observed is provided in Table 2.

The most frequently reported adverse reaction to ICIs was elevated transaminase levels, occurring in 65.8% (25 out of 38) of patients. This was followed by diarrhea or colitis, reported in 23.7% (9 out of 38) of patients, endocrine disorders 21.1% (8 out of 38), and cutaneous toxic effects in 15.8% (6 out of 38). Notably, patients who developed diarrhea or colitis exhibited a significantly higher probability of achieving CR or PR compared to those with SD or PD. Patients with CR and PR as their best radiological response had diarrhea occurrence rates of 50% [95% CI: 15.7–84.3] and 35.7% [95% CI: 12.8–64.9], respectively. However, the rates of diarrhea in patients with SD and PD as the best radiological response were 0% [95% CI: 0–52.2 and 95% CI: 0–28.5], respectively.

### 2.3. cfDNA Levels

**Figure 2:**
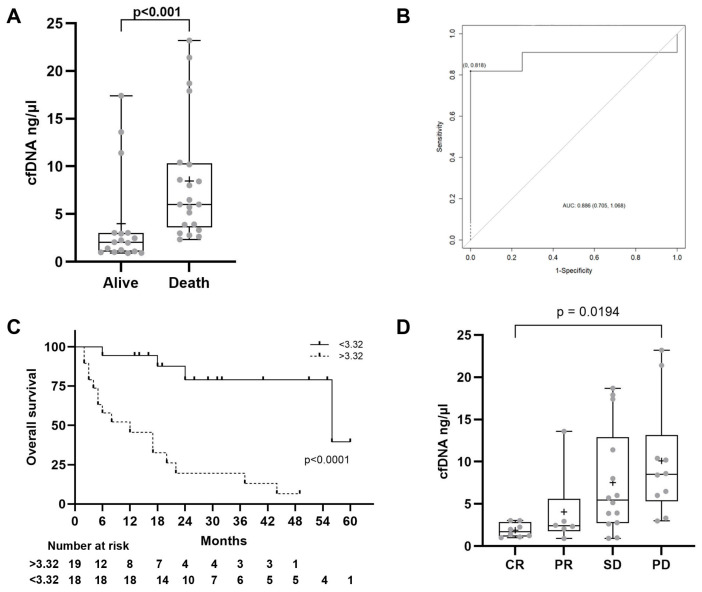
cfDNA levels. (**A**) Basal cfDNA levels in alive vs. death patients. The statistical method used was the *t*-test. (**B**) ROC curve distinguishing patients with more than 3.32 ng/µL. (**C**) Kaplan–Meier curve of overall survival for HCC patients stratified by baseline cfDNA levels; *p*-value from the log-rank test. (**D**) Basal cfDNA levels in patients with CR, PR, SD, and PD. CR: complete response, PR: partial response, SD: stable disease, PD: progressive disease. The statistical method used was one-way ANOVA followed by Tukey’s multiple comparisons test.

In terms of survival, cfDNA levels at diagnosis were significantly lower in patients who were alive at the end of the follow-up period than in those who died during follow-up. The mean (standard deviation) cfDNA levels were 4 (5) ng/μL for survivors vs. 8.5 (6.4) ng/μL for non-survivors (HR 1.134, *p* < 0.001) (Figure 2A). Baseline levels of cfDNA and cfDNA after three months of ICI treatment are summarized in Table 3.

To assess the predictive value of baseline cfDNA for OS, receiver operating characteristic (ROC) curves were generated. With a cut-off of 3.32 ng/μL, basal cfDNA showed good accuracy in discriminating patients with CR or PD as their best radiological response, achieving an area under the curve (AUC) of 0.886 with a sensitivity of 81.82% and specificity of 87.5% (Figure 2B). Kaplan–Meier survival analysis indicated that patients with more than 3.32 ng/μL of basal cfDNA had significantly higher mortality compared to those with levels below this threshold (median survival time 26 months vs. 8 months, *p* = 0.0001) based on Kaplan–Meier’s survival analysis (Figure 2C).

Regarding the relationship between cfDNA and radiological response, we evaluated the potential value of cfDNA levels to predict the efficacy of ICI therapy in HCC patients. Our analysis revealed that basal cfDNA levels were significantly different across radiological response categories, with lower levels in patients who showed a radiological response compared to those with SD or PD [mean (standard deviation) values for CR, PR, SD, and PD: 1.88 (0.83), 4.68 (5), 7.53 (6.30), and 9.26 (7.13) ng/μL, respectively] (*p* = 0.0194) (Figure 2D). Similarly, cfDNA levels measured three months after initiation of ICI treatment were also significantly different among patients with different radiological responses: mean (standard deviation) levels were 3.02 (2.26), 3.27 (2.80), 13.78 (23.00), and 11.36 (13.02) ng/μL for CR, PR, SD, and PD, respectively (*p* = 0.026).

### 2.4. ctDNA Levels

In alignment with the baseline cfDNA findings, univariate Cox proportional hazards analysis revealed that higher baseline ctDNA levels were significantly associated with reduced OS (HR 1.080, CI 1.010–1.154, *p* = 0.024). Similar associations were observed for ctDNA levels measured three months after initiation of immune checkpoint inhibitor (ICI) therapy (HR 1.079, 95% CI 1.003–1.161, *p* = 0.041) (Table 3 and Supplementary Table S2).

**Figure 3:**
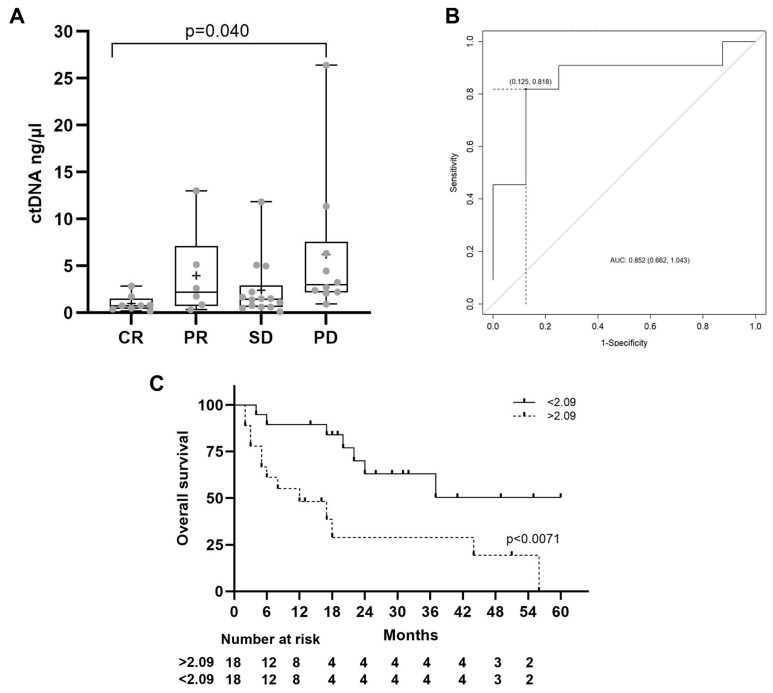
ctDNA levels. (**A**) Basal ctDNA levels in patients with CR, PR, SD, and PD. The statistical method used was the one-way ANOVA followed by Tukey’s multiple comparisons test. (**B**) ROC curve distinguishing patients with more than 2.09 ng/µL. (**C**) Kaplan–Meier curve of overall survival for HCC patients stratified by baseline ctDNA level; *p*-value from the log-rank test. CR: complete response, PR: partial response, SD: stable disease, PD: progressive disease.

As in the case of cfDNA, baseline ctDNA levels were evaluated as potential markers of response to ICIs. Significant differences were noted in baseline ctDNA levels among patients exhibiting CR, PR, SD, or PD [0.99 (0.88), 4.68 (4.92), 2.42 (3.11), and 5.68 (7.52) ng/μL, respectively] (*p* = 0.04) (Figure 3A).

Three months post-ICI initiation, ctDNA levels remained indicative of therapeutic response, being significantly lower in patients showing CR or PR [2.2 (3.1) ng/μL] compared to those with SD [6.4 (7.8) ng/μL] or PD [4.5 (4.8) ng/μL] (*p* = 0.046). Furthermore, statistically significant differences were identified in the changes of ctDNA levels across these response groups after three months of ICI treatment (mean (standard deviation) for CR, PR, SD, PD: 0.4 (2.2), −1.2 (4.5), 3.9 (5.1), −1.1 (8.5), respectively, *p* = 0.047).

For predictive assessment, baseline ctDNA levels were evaluated for their ability to distinguish between patients achieving CR versus PD. A threshold of 2.09 ng/μL was determined to optimize sensitivity (81.82%) and specificity (87.5%), with an area under the curve (AUC) of 0.852 in the receiver operating characteristic (ROC) analysis. Kaplan–Meier survival analysis demonstrated a median survival time of 10 months for patients with ctDNA levels exceeding 2.09 ng/μL, versus 24 months for those with levels below this cutoff (*p* = 0.0071) (Figure 3C).

### 2.5. Somatic Mutation Analysis

The 228T *TERT* promoter mutation was determined in cfDNA using ddPCR at baseline and three months post-initiation of treatment in 38 patients treated with ICIs. The mutation was present in 98% of samples, with a frequency ranging from 0.0007% to 60%. No correlation was identified between the percentage of *TERT* promoter mutation and PFS or OS.

Baseline ctDNA profiling was assessed by the Onco-500 TruSight panel on basal samples from 21 patients. The mean and median number of somatic mutations per megabase (TMB) in the study population were 58.92 and 52.78 mutations/Mb, respectively. A high TMB, defined as 10 or more mutations/Mb, was observed in the majority of patients (18/21). TMB did not show significant associations with survival outcomes or responses to ICIs.

**Figure 4:**
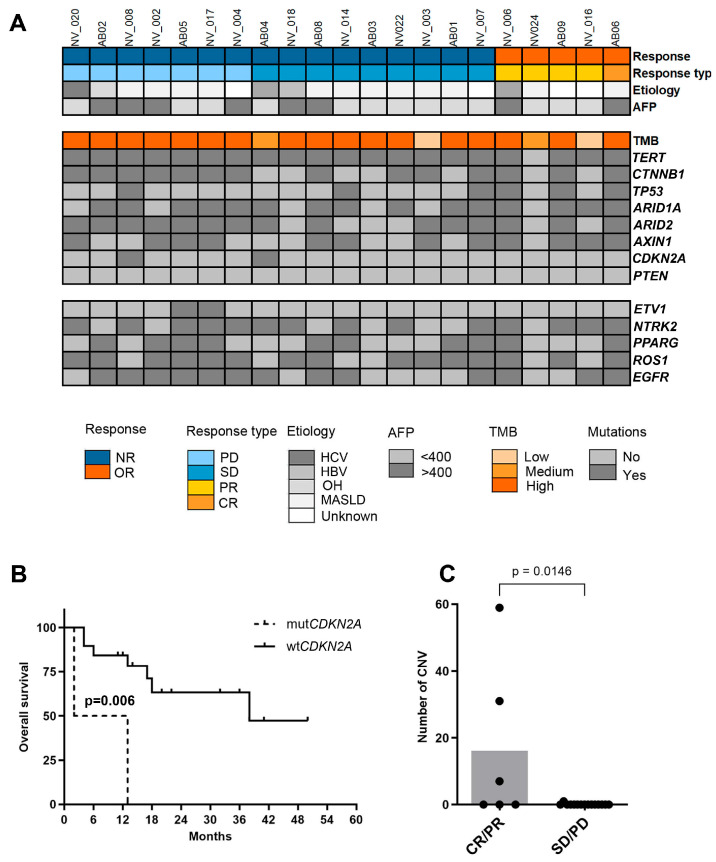
(**A**) Genomic ctDNA profiling of HCC patients treated with ICI. (**B**).Kaplan–Meier curves of overall survival according to the presence or absence of *CDKN2A* mutations. (**C**) Number of copy number variations (CNVs), the statistical method used was the *T*-test. AFP: alpha-fetoprotein, TMB: tumor mutational burden, NR: no response, OR: objective response, CR: complete response, PR: partial response, SD: stable disease, PD: progressive disease, HCV: hepatitis C virus, HBV: hepatitis B virus, OH: alcohol, MASLD: metabolic-associated steatotic liver disease, CNV: copy number variation.

In the 21 plasma samples analyzed, pathological ctDNA mutations were identified in several genes: *CTNNB1* in 14 out of 21 patients (66%) with a total of thirty-eight mutations, *TP53* in 6 out of 21 patients (29%) with sixty-four mutations, *ARID1A* in 15 out of 21 patients (71%) with seventy-three mutations, *ARID2* in 15 out of 21 patients (71%) with eighty-five mutations, *AXIN1* in 12 out of 21 patients (57%) with thirty-five mutations, and *CDKN2A* in 2 out of 21 patients (9.52%) with four mutations (Figure 4A, Supplementary Table S3).

The most heavily mutated genes in our cohort were *ETV1* with 700 mutations in 2 out of 21 patients, *NTRK2* with 432 mutations in 14 out of 21 patients, *PPARG* with 382 mutations in 10 out of 21 patients, *ROS1* with 327 mutations in 15 out of 21 patients, and *EGFR* with 276 mutations in 13 out of 21 patients (Supplementary Table S3).

The presence of pathological mutations in the commonly mutated genes in HCC, such as *TERT*, *CTNNB1*, *TP53*, *AXIN1*, *ARID1A*, or *ARID2*, did not significantly influence OS. Interestingly, patients with mutations in the *CDKN2A* gene exhibited significantly reduced survival rates, with a median survival of 7.5 months, compared to a median of 38 months in patients without *CDKN2A* mutations (*p* = 0.006) (Figure 4B).

No significant differences in outcomes were observed based on the presence of pathological mutations in *CTNNB1*. Nevertheless, *CTNNB1* pathogenic mutations were present in 100% of patient exhibiting PD as the best radiological outcome (Supplementary Table S3).

### 2.6. Copy Number Variation

Patients presenting PD as their best radiological response had significantly fewer copy number variations (CNVs) compared to those exhibiting a radiological response, either CR or PR. Specifically, patients showing a radiological response showed 97 CNVs (3 patients) whereas those patients without radiological response exhibited only one CNV in 1 patient. The mean (standard deviation) CNVs were 0.16 (0.41) in patients with SD or PD and 16.1 (24.17) in patients with CR or PR (*p* < 0.05) (Figure 4C).

### 2.7. Cytokine Levels

We assessed the levels of 24 cytokines in plasma samples collected at baseline and three months after initiating ICI treatment and analyzed their correlation with radiological response as per mRECIST and OS.

**Figure 5:**
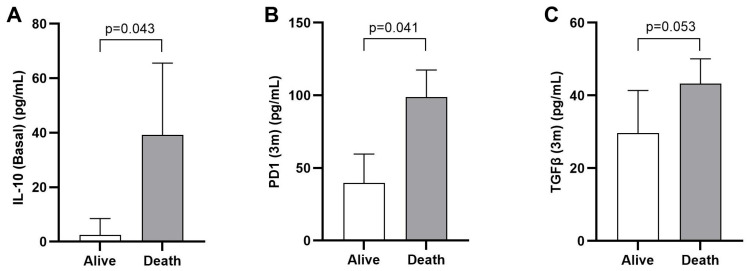
Cytokine levels. (**A**) Basal levels of IL10 in patients alive and death. (**B**,**C**) Levels of PD1 and TGF-β, respectively, measured after three months (3 m) of receiving ICI treatment according to status (Alive vs. Death). The statistical method used was the *t*-test.

Compared to patients who remained alive, we found higher levels of basal IL10 among patients who died [mean (standard deviation) 2.5 (6) vs. 39.2 (120.9) pg/mL, *p* = 0.043]. Elevated PD-1 levels after three months of treatment were also associated with a poorer prognosis in terms of OS [mean (standard deviation) 98.8 (85.8) pg/mL vs. 39.7 (19.8) pg/mL, *p* = 0.041]. Although not statistically significant, higher TGF-β levels at three months post-treatment initiation showed a trend toward association with worse outcomes [mean (standard deviation) 43.2 (28.2) pg/mL vs. 29.6 (46.9) pg/mL, *p* = 0.053] (Figure 5).

In the univariate analysis, higher levels of CTLA4 (cytotoxic T lymphocyte antigen 4), IFN-Beta, IL-1 Beta, IL6, PD1 (programmed cell death 1), and LAG-3 at three months of starting ICI treatment were associated with poor survival (Supplementary Table S4). Regarding radiological response, no significant differences were observed in cytokine levels.

## 3. Discussion

Immunotherapy has significantly impacted survival expectations for patients living with advanced HCC. However, not all patients derive equal benefit from this therapeutic approach, and reliable biomarkers for patient selection a priori are currently lacking [6,7]. Identifying patients unlikely to benefit from immune checkpoint inhibitors (ICIs) is crucial to avoid adverse events, ineffective treatments, and allow for the timely introduction of potentially more effective therapies. This highlights an urgent need for non-invasive biomarkers in this clinical setting [8].

In this prospective study, we investigated the association between levels of plasma biomarkers (such as cfDNA, ctDNA, and cytokines) and the response to immune checkpoint inhibitors in advanced HCC.

cfDNA has been thoroughly explored as a non-invasive biomarker in cancer [19,20,21]. Our research indicates that higher cfDNA and ctDNA levels are significantly associated with poorer OS and a lack of radiological response to ICI therapy. These findings suggest that a simple quantification of cfDNA/ctDNA could predict clinical outcomes and monitor dynamic changes during ICI treatment.

Previous studies have shown that cfDNA concentrations are higher in HCC patients compared to those with chronic hepatitis and healthy controls [19,21], associating them with early recurrence and poor survival post-surgical resection [21]. Our results are consistent with recent findings by Matsumae et al., showing in a similar study performed in HCC patients treated with atezolizumab/bevacizumab, that elevated cfDNA levels correlate with lower ORR and shorter PFS and OS [22].

We observed that cfDNA and ctDNA levels were significantly higher in patients not showing a radiological response to ICI treatment and in those with poorer OS. These differences were noted at both baseline and after three months, supporting the potential utility of cfDNA/ctDNA quantification in predicting clinical outcomes and monitoring treatment dynamics.

*TERT* promoter mutations have been associated with poor prognosis after treatment; however, the majority of these studies comprised patients with early-stage HCC [23]. We aimed to assess its potential role in the advanced HCC setting by ddPCR, a more rapid and affordable sequencing alternative that we validated in a previous study [21]. As *TERT* has been described as a gatekeeper in HCC development [24], a high percentage of patients with mutations in this gene in our advanced HCC cohort was expected. The C228T *TERT* mutation was found in 98% of patients, but, unfortunately, we did not find a significant correlation between the percentage of this mutation in cfDNA and radiological response or OS [25,26].

The role of *TERT* mutations in cancer has been a subject of controversy, as described in previous studies [25,26]. Li H et al. reported that *TERT* mutations were associated with a higher tumor mutational burden (TMB), suggesting increased responsiveness to immunotherapy. Specifically, they found that prognosis was better in patients with *TERT* mutations treated with anti-CTLA-4 antibodies, whereas the prognosis was similar between those with and without TERT mutations when treated with anti-PD-1/PD-L1 therapies [25]. This variability in treatment response could explain our findings.

Regarding ctDNA mutations, our findings align with previous research on HCC tissue [27], identifying frequent mutations in the *TERT* promoter (98% of patients), followed by *ARID1A* (71%), *CTNNB1* (67%), *AXIN1* (57%), *TP53* (29%), and *CDKN2A* (10%).

The WNT/β-catenin pathway, which is activated by mutations in the *CTNNB1* gene, was found to be associated with a lower disease control rate, a shorter median PFS, and lower median OS as compared to WNT wild-type [13,28,29]. Consequently, we focused on *CTNNB1* mutations. Although *CTNNB1* mutations can activate the Wnt pathway, leading to T-cell exhaustion and innate resistance to immune checkpoint inhibitors [30], we found no significant differences in radiological response or OS. These findings are consistent with those reported by Matsumae et al., who also observed no difference in treatment response or patient prognosis based on the presence or absence of *CTNNB1* mutations [22]. These could potentially be attributed to the ability of VEGF blockade to overcome Wnt/β-catenin resistance, particularly in patients treated with atezolizumab/bevacizumab [11,12]. VEGF blockade is known to reduce immunosuppressive cell populations, increase cytotoxic T cell infiltration, and enhance tumor recognition and cancer cell death [31].

Interestingly, patients with *CDKN2A* mutations in our cohort were more likely to experience disease progression during treatment and, consequently, had significantly poorer OS, consistent with observations in other cancers, such as non-small cell lung cancer (NSCLC) [32].

Copy number variations (CNVs) were significantly lower in patients with PD, a pattern seen in NSCLC [33]. However, conflicting data in hepatobiliary cancers deserve further study to clarify CNVs’ role as a biomarker in HCC [34].

Furthermore, a substantial body of evidence has emerged indicating a correlation between immune-related adverse events (irAEs) and immunotherapy efficacy in various tumor types, including HCC [35,36]. Ng et al. described how patients who experienced irAEs had superior OS, PFS, ORR, and DCR (disease control rate) [36]. In our study, patients who experienced irAEs such as diarrhea or colitis had higher rates of complete or partial response compared to those with stable or progressive disease, aligning with published data [35,36].

Cytokine levels, which are critical to immune responses, were evaluated at baseline and three months after treatment initiation. While PD-L1 expression is a well-studied predictor of ICI outcomes in several cancer types [37,38,39,40], its value in HCC remains unclear [3,9]. Our study found that neither baseline plasma PD-1 nor PD-L1 levels, either basal or after three months of ICI treatment, could effectively differentiate responders. However, elevated PD-L1 levels measured after three months of ICI treatment correlated with worse prognosis.

Additionally, elevated baseline IL-10 levels were associated with poorer survival, supporting its role in disease progression and immune suppression [41,42]. Interleukin-10 (IL-10) is a multifaceted cytokine produced by a diverse array of cells and plays a significant role in various physiological processes. The immunosuppressive properties of IL-10 have been well documented in several studies, including some on HCC, which show a correlation between elevated IL-10 serum levels and advanced disease stages [41,42,43]. Furthermore, higher levels of TGF-β were associated with poor outcomes. Similar findings were reported by Feun LG et al., who reported that pembrolizumab increased both OS and PFS rates in HCC patients with low baseline TGF-β levels [44]. Our study has certain limitations, including a small, single-center sample size and the need for validation in larger, multi-institutional cohorts before any recommendations can be made. Despite these limitations, our proposed biomarkers are easy to implement and could enhance clinical decision-making for advanced HCC once validated.

In conclusion, while immunotherapy and ICIs, in particular, have revolutionized advanced HCC treatment, identifying patients who will benefit remains challenging. Our study proposes practical, reproducible biomarkers that, pending validation, could significantly improve precision in treatment selection and outcomes for patients with advanced HCC.

## 4. Materials and Methods

### 4.1. Study Design and Participants

Patients were prospectively recruited at the Liver Unit, Hospital Universitari Vall d’Hebron. Plasma samples from 38 unresectable HCC patients eligible to receive ICI were prospectively collected. Treatments applied were as follows: Nivolumab monotherapy (n = 15), atezolizumab/bevacizumab combination (n = 19), durvalumab/tremelimumab combination (n = 2), lenvatinib/pembrolizumab combination (n = 1) or pembrolizumab monotherapy (n = 1). ORR and OS were assessed using the mRECIST criteria.

Plasma samples from all patients were collected within the 24–48 h before starting immunotherapy and three months after the beginning of the treatment. From these 76 samples, cytokine levels by ELISA, and cfDNA levels, ctDNA levels, and TERT percentage mutation by ddPCR were analyzed. Onco-500 TruSight was performed in basal samples (plasma and PBMC) from 21 of these patients, due to limited ctDNA quantity or quality (Supplementary Figure S1).

The institutional ethical review board approved the protocol (PR(AG)194/2015), and all patients gave their written informed consent before inclusion.

### 4.2. Plasma Collection

Peripheral venous blood was collected at least within the 24–48 h prior to immunotherapy treatment in a Lithium Heparin Tube (BD Biosciences, Franklin Lakes, NJ, USA) and processed within 4 h of collection. Plasma was collected after centrifugation at 1500× *g* for 15 min at 4 °C and was immediately stored at −80 °C.

### 4.3. cfDNA and ctDNA Extraction and Quantification

Circulating DNA was isolated from 1 mL of plasma using the MagMAX™ Cell-Free DNA Isolation Kit (Thermo Fisher, Waltham, MA, USA). Blood DNA was isolated using the QIAamp DNA Mini Kit (Qiagen, Hilden, Germany) according to the manufacturer’s recommendations. Purified DNA concentration was measured by fluorometric quantitation using Qubit (Thermo Fisher, Waltham, MA, USA).

ctDNA concentration was estimated using Agilent D1000 ScreenTape (Agilent Technologies, Santa Clara, CA, USA) and analyzed using TapeStation Analysis Software A.02.01 SR1, considering ctDNA fragments smaller than 200 bp.

### 4.4. Droplet Digital PCR (ddPCR)

C228T TERT mutation was analyzed by the QuantStudio 3D Digital PCR System (Thermo Fisher, Waltham, MA, USA), with TaqMan primers and probes also from Thermo Fisher (Hs000000092_rm). The analysis of wild-type and mutated alleles was performed using QuantStudio 3D AnalysisSuite Cloud Software v3.0 from Thermo Fisher.

### 4.5. Cytokines and Growth Factor Determination

Multiplex ELISA was performed using a custom-made panel of 24 cytokines and growth factors (BTLA, CD137 (4-1BB), CD152 (CTLA4), CD27, CD28, CD80, GITR, HGF, HVEM, IFN beta, IFN gamma, IL-1 beta, IL-10, IL-12/IL-23p40, IL-21, IL-6, IP-10 (CXCL10), LAG-3, MCP-1 (CCL2), PD-1, PD-L1, PD-L2, TIM-3, TNF alpha) in plasma samples according to the manufacturer’s guidelines (ProcartaPlex Multiplex Immunoassay, Thermo Fisher, Waltham, MA, USA).

Data were acquired using a MagPix Luminex XMAP (Thermo Fisher, Waltham, MA; USA) and analyzed with the ProcartaPlex Analysis App from Thermo Fisher.

PD-1, PD-L1, and TGF-β plasma levels were also measured with an ELISA kit with higher sensitivity (Thermo Fisher, Waltham, MA, USA). Data were acquired with a Varioskan Lux Reader (Thermo Fisher, Waltham, MA, USA) and analyzed by Skanlt Software v7.1 for Microplate Readers from Thermo Scientific.

### 4.6. Trusight

The quantity and integrity of the ctDNA and genomic DNA were evaluated with the Qubit dsDNA HS DNA Kit (Thermo Fisher Scientific) and agarose gels, respectively. Sequencing libraries were prepared following the TruSight Oncology 500 ctDNA Reference Guide (Document # 1000000092559 v00) with the corresponding kit (Illumina Inc. TruSight Oncology 500 ctDNA Kit (Illumina, San Diego, CA, USA)).

The genomic library was prepared using End Repair A-Tailing Master Mix, input DNA was blunt-ended, and the 3′ ends were A-tailed. Then, UMI1 adapters were ligated to DNA fragments. Library fragments were amplified and hybridized to the specific 523 genes targeted by the TruSight Oncology 500 ctDNA panel. Finally, enriched libraries were amplified and sequenced on an Illumina NovaSeq6000.

FastQ files resulting from library sequencing were aligned to the reference genome (hg38) using BWA version 0.7.17-r1188. The resulting aligned BAM files were sorted and processed for PCR duplicates removal, base quality score recalibration, and indel realignment using samtools 1.9, the Genome Analysis Tool Kit (GATK) version 4.1.4.1, and picard version 2.17.0. Somatic and germline single nucleotide variants and small indels, as well as copy number variations, were called using GATK’s Haplotype Caller version 4.1.4.1 [45]. Structural variation was called using Delly2 version 0.8.1 [46]. Annotation of the called genetic variants was completed using Annovar version 2018-04-16 [47].

### 4.7. Statistical Analysis

Quantitative variables were described using mean and standard deviation, and qualitative variables as absolute frequency and percentage. To compare the means of two different groups with one categorical variable that follows a normal distribution, a *t*-test was performed. To compare means of more than two groups, a one-way ANOVA test was performed, followed by post-hoc Tukey’s multiple comparisons test to assess differences between the means of all the possible pairs.

Associations between cfDNA or cytokine levels and clinical outcomes were assessed using the nonparametric Mann–Whitney U-test. The Kaplan–Meier method was used to estimate survival and recurrence curves according to cfDNA and cytokine levels. Comparison of the survival curves between groups was performed using the log-rank (Mantel–Cox) test. The diagnostic performance of cfDNA and cytokines to predict survival was assessed using the area under the curve (AUC) and its respective 95% confidence interval of the receiver operating characteristic (ROC) curve. The optimal cut-off of ctDNA and cytokines was defined using ROC curves, choosing the one with the best accuracy according to the Youden index. Significance was considered as two-sided *p* values < 0.05. Data analysis was performed with the statistical package “R” (R version 4.3.0 (2023-04-21 ucrt), Copyright © 2015 The R Foundation for Statistical Computing).

## Figures and Tables

**Table 1:** Clinicopathological characteristics of HCC patients.

Demographic	Cases (n = 38)	
	Clinicopathological characteristics	
Gender, n (%)	Male	31 (81.6%)
Age, median (range)		68.7 (62.1–76.7)
Etiology, n (%)	HCV	21 (55.3%)
	MASLD	5 (13.2%)
	HBV	2 (5.3%)
	ALD	3 (7.9%)
	Others	1 (2.6%)
	No liver disease	6 (15.8%)
BCLC, n (%)	BCLC B	8 (21.1%)
	BCLC C	30 (78.9%)
ECOG, n (%)	ECOG 0	32 (84.2%)
	ECOG 1	6 (15.8%)
Child Pugh, n (%)	A	32 (84.2%)
	B	6 (15.8%)
Previous treatment, n (%)	Locoregional or surgery	22 (57.89%)
	TKI	17 (44.7%)
	No previous treatment	10 (26.3%)
ICI therapy, n(%)	Nivolumab	15 (39.5%)
	Atezolizumab/Bevacizumab	19 (50%)
	Durvalumab/Tremelimumab	1 (2.6%)
	Lenvatinib/Pembrolizumab	1 (2.6%)
	Pembrolizumab	1 (2.6%)
	Laboratory values	
AFP (ng/dL), median (range)		17.9 (4.5–1082.3)
Bilirubin (mg/dL), median (range)		0.8 (0.6–1.2)
Albumin (g/L), median (range)		4 (3.7–4.2)
Platelet count (10^9^/L), median (range)		186 (102–245)
INR		1 (1–1.1)
AST (UI/L)		51 (39–2.81)
ALT (UI/L)		39 (27.5–55.5)
	Radiological response	
Best Radiological response mRECIST, n (%)	Complete response (CR)	8 (21.1%)
	Partial response (PR)	5 (13.2%)
	Stable disease (SD)	14 (36.8%)
	Progressive disease (PD)	11 (28.9%)
Events, n (%)	Deceased	21 (55.3%)
Time to radiological progression, median months (range)		14 (2.5–25.4)
Survival, median months (range)		24 (2.3–45.6)
Follow-up, median months (range)		16.5 (2–60)

HCV: hepatitis C virus, MASLD: metabolic-associated steatotic liver disease, HBV: hepatitis B virus, ALD: alcohol-related liver disease, TKI: tyrosine kinase inhibitor, ICI: immune checkpoint inhibitors, AFP: alpha-fetoprotein, mRECIST: Modified Response Evaluation Criteria in Solid Tumors.

**Table 2:** Immune-related adverse events.

Immune-Related Adverse Events (irAEs)		
irAEs, n (%)		19 (50%)
Number of AEs, n (%)	0	19 (50%)
	1	11 (28.9%)
	2	5 (13.2%)
	3	3 (7.9%)
Transaminases increase, n (%)		25 (65.8)
Diarrhea or colitis, n (%)		9 (23.7%)
Endocrine disorders, n (%)		8 (21.1%)
Dermatological, n (%)		6 (15.8%)

**Table 3:** cfDNA and ctDNA levels (ng/µL), mean (SD).

	cfDNA		ctDNA	
**Radiological Response**	**Basal**	**3 m**	**Basal**	**3 m**
CR	1.9 (0.8)	3 (2.3)	1(0.9)	1.4 (1.9)
PR	7.5 (6.3)	13.8 (23)	4.7 (4.9)	3.5 (4.4)
SD	4.7 (5)	3.3 (2.8)	2.4 (3.1)	6.4 (7.8)
PD	9.3 (7.1)	11.4 (13)	5.7 (7.5)	4.5 (4.8)
**Status**	**Basal**	**3 m**	**Basal**	**3 m**
Alive	4 (5)	9.5 (21.7))	2.4 (3.2)	4 (6)
Death	8.5 (6.4)	9.3 (9.9)	4.1 (6)	4.7 (5.8)

## Data Availability

Raw sequencing data from samples included in this article will be openly available upon publi-cation via GEO of the NCBI (Accession number GSE237791).

## Supplementary Materials

The following supporting information can be downloaded at: https://www.mdpi.com/article/10.3390/ijms26062794/s1.

## Abbreviations

The following abbreviations are used in this manuscript:

AFP	Alpha-fetoprotein
cfDNA	Cell-free DNA (cfDNA)
CNVs	Copy number variations
CR	Complete response
ctDNA	Circulating tumoral DNA
CTLA4	Cytotoxic T-Lymphocyte Antigen 4
GGT	Gamma-glutamyl transferase
HCC	Hepatocellular carcinoma
ICIs	Immune checkpoint inhibitors
irAEs	Immune-related adverse events
mRECIST	Modified Response Evaluation Criteria in Solid Tumors
ORR	Objective radiological response
PD	Progressive disease
PD-L1	Programmed cell Death-ligand 1
PFS	Progression-free survival
PR	Partial response
TMB	Tumor mutational burden
SD	Stable disease
OS	Overall survival
