# Supplementary material for: Harnessing Plasma Biomarkers to Predict Immunotherapy Outcomes in Hepatocellular Carcinoma: The Role of cfDNA, ctDNA, and Cytokines
Source: Int J Mol Sci. 2025 Mar 20;26(6):2794. doi: 10.3390/ijms26062794 (PMC11942713; doi:10.3390/ijms26062794)
Supplement: Supplementary file 1 [file ijms-26-02794-s001.zip › Supplentary Table S1-S4.pdf]

Supplementary material

Supplementary Figures

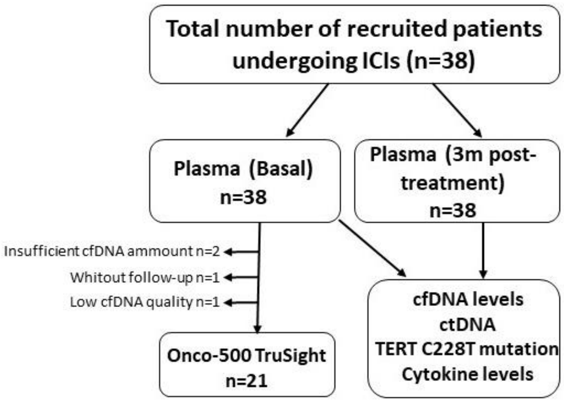

Supplementary Figure S1. Flow diagram of the study design. cfDNA: cell-free DNA, TERT: telomerase reverse transcriptase.

Supplementary tables

Supplementary Table S1: Analysis of basal analytics by survival status (Median [CI])

Variable	Alive	Death	p-Value
AST (UI/L)	52 [37,77]	50 [40,81]	0.769
ALT (UI/L)	31 [27,51]	44 [30,57]	0.769
GGT (UI/L)	80 [40,134]	189 [92, 282]	**0.018***
Alkaline phosphatase (UI/L)	119 [94, 155]	147 [126, 242]	0.076
Bilirrubin (total) mg/dL	1.2 [0.7,2]	0.8 [0.6, 0.9]	0.06
Bilirrubin (direct) mg/dL	0.3 [0.2, 0.7]	0.3 [0.2, 0.3]	0.258
Tirotropin (mU/L)	2.8 [1, 1296]	3.4 [1.6, 1060.4]	0.424
Albumin (g/L)	4 [3.8, 4.2]	3.9 [3.5, 4.2]	0.714
Creatinin (mg/dL)	0.7 [0.6, 0.9]	0.8 [0.7, 0.9]	0.394
Platelets (x10^9/L)	188 [89, 208]	186 [149, 276]	0.445
Hemoglobin (g/dL)	14 [13.3, 15]	12.5 [11.6, 13.6]	**0.03***
AFP (ng/mL)	5.3 [4.4, 262.1]	37 [6.4, 17459.4]	0.13
AFP 3m (ng/mL)	3.9 [2, 7.2]	157.5 [8.7, 7875]	**0.008***
AFP (Difference 0-3m) ng/mL	-2.1 [-10.7, 0]	3.7 [-0.2, 726.3]	**0.004***

AST: aspartate aminotransferase, ALT: alanine transaminase, GGT: gamma glutamyltransferase, AFP: Alpha fetoprotein, *statistically significant

Supplementary Table S2: Univariate Cox analysis of independent risk factors of survival (Biochemistry and cf/ctDNA)

VARIABLE	HR	(95%CI)	p-Value
Etiology	1.00	(0.77; 1.28)	0.99
AST (UI/L)	1.01	(0.99; 1.02)	0.09
ALT (UI/L)	1.00	(0.98; 1.02)	1.02
**GGT (UI/L)**	**1.00**	**(1.00; 1.00)**	**0.005***
Bilirrubin (total) mg/dL	0.46	(0.19;1.09)	0.079
Albumin (g/L)	1.13	(0.40;3.17)	0.814
Hemoglobin (g/dL)	0.80	(0.63;1.05)	0.055
**Platelets (x10^9/L)**	**1.00**	**(1.00; 1.007)**	**0.049***
AFP(ng/mL)	1.00	(1.00;1.00)	0.118
**cfDNA**	**1.13**	**(1.05;1.21)**	**<0.001***
cfDNA 3m	1.01	(0.99;1.03)	0.271
**ctDNA**	**1.08**	**(1.01; 1.15)**	**0.024***
**ctDNA 3m**	**1.07**	**(1.00;1.16)**	**0.041***

AST: aspartate aminotransferase, ALT: alanine transaminase, GGT: gamma glutamyltransferase, AFP: Alpha fetoproteïna, cfDNA: Cell-free DNA, ctDNA: circulating tumoral DNA. *statistically significant

Supplementary Table S3: Mutations ctDNA

Supplementary Table S4: Univariate Cox analysis of independent risk factors of survival (cytokines)

VARIABLE	HR	(95%CI)	p-Value
BTLA	1.00	(1.00;1.00)	0.569
BTLA 3m	1.00	(1.00;1.00)	0.511
CD27	0.99	(0.99;1.01)	0.555
CD27 3m	1.00	(0.99;1.00)	0.194
CD28	1.00	(0.99;1.00)	0.290
**CD28 3m**	**1.00**	**(1.00;1.00)**	**0.038***
CD80	1.00	(0.99;1.00)	0.560
CD80 3m	1.00	(0.99;1.00)	0.282
CD137	1.00	(0.99;1.00)	0.361
CD137 3m	1.00	(0.99;1.00)	0.187
CTLA4	1.00	(0.99;1.00)	0.168
**CTLA4 3m**	**1.00**	**(0.99;1.00)**	**0.0003***
GITR	1.00	(0.99;1.00)	0.485
GITR 3m	1.00	(0.99;1.00)	0.086
HGF	1.00	(0.99;1.00)	0.573
HGF 3m	1.00	(0.99;1.00)	0.337
HVEM	1.00	(0.99;1.00)	0.37
HVEM 3m	1.00	(0.99;1.00)	0.212
IFNβ	1.00	(0.99;1.00)	0.322
**IFNβ 3m**	**1.00**	**(1.00;1.00)**	**0.041***
IFNγ	1.00	(0.99;1.00)	0.342
IFNγ 3m	1.00	(1.00;1.00)	0.153

IL1β	1.00	(0.99;1.00)	0.576
**IL1β 3m**	**1.00**	**(1.00;1.00)**	**0.024***
IL6	1.00	(1.00;1.00)	0.568
**IL6 3m**	**1.00**	**(1.00;1.00)**	**0.018***
IL10	1.00	(0.99;1.00)	0.299
IL10 3m	1.00	(0.99;1.01)	0.192
IL12	1.00	(0.99;1.00)	0.328
IL12 3m	1.02	(0.94;1.10)	0.549
IL21	1.00	(0.99;1.00)	0.328
IL21 3m	1.00	(0.99;1.00)	0.37
IP10	1.00	(0.99;1.01)	0.052
IP10 3m	1.00	(0.99;1.00)	0.064
LAG3	1.00	(0.99;1.00)	0.179
**LAG3 3m**	**1.00**	**(1.00;1.00)**	**0.002***
MCP1	1.00	(0.99,1.00)	0.758
MCP1 3m	0.99	(0.99;1.00)	0.988
PD1	1.00	(0.99;1.00)	0.202
**PD1 3m**	**1.01**	**(1.00;1.01)**	**<0.001***
PDL1	1.00	(0.92;1.07)	0.99
PDL1 3m	0.99	(0.95;1.03)	0.871
PDL2	1.00	(1.00;1.00)	0.61
PDL2 3m	1.00	(0.99;1.00)	0.967
TIM3	0.99	(0.99;1.00)	0.838
TIM3 3m	1.00	(0.99;1.01)	0.207
TNFα	1.00	(0.99;1.01)	0.426
TNFα 3m	1.00	(0.99;1.00)	0.172
TGFβ	0.99	(0.98;1.01)	0.82
TGFβ 3m	1.00	(0.99;1.01)	0.67

BTLA: B- and T-lymphocyte attenuator, CD: Cluster of differentiation, CTLA4: Cytotoxic T-Lymphocyte Antigen 4, GITR: Glucocorticoid-induced tumor necrosis factor related protein, HGF: Hepatocyte growth factor, HVEM: herpes virus entry mediator, IFNβ: Interferon beta, IFNγ: interferon gamma, IL: interleukine, IP10: Interferon gamma-induced protein 10, LAG3: Lymphocyte activation gene 3 protein, MCP1: Monocyte chemoattractant protein-1, PD1: Programmed Cell Death Protein 1, PD-L1: Programmed Cell Death Protein Ligand 1, TIM3: T cell immunoglobulin mucin, TNF: tumor necrosis factor. *statistically significant
